# A photo-crosslinkable hyaluronic acid–collagen hydrogel maintains epithelial integrity and supports intestinal organoid stemness

**DOI:** 10.1093/rb/rbag171

**Published:** 2026-08-07

**Authors:** Yeonoh Cho, Se Yeon Park, Dong-Wook Han, Hyun Jong Lee, Jong Hun Lee

**Affiliations:** Department of Food Science and Biotechnology, Gachon University, Seongnam-si 13120, Republic of Korea; School of Chemical, Biological and Battery Engineering, Gachon University, Seongnam-si 13120, Republic of Korea; Department of Cogno-Mechatronics Engineering, Pusan National University, Busan 46241, Republic of Korea; School of Chemical, Biological and Battery Engineering, Gachon University, Seongnam-si 13120, Republic of Korea; Department of Food Science and Biotechnology, Gachon University, Seongnam-si 13120, Republic of Korea

**Keywords:** organoids, ECM, hyaluronic acid–collagen hydrogel, photo-crosslinking, stemness and epithelial integrity

## Abstract

Organoids provide physiologically relevant platforms for disease modeling and drug screening, but their performance depends critically on the extracellular matrix (ECM). Matrigel, the current standard, suffers from undefined composition, batch-to-batch variability and limited tunability. We engineered a photo-crosslinkable hyaluronic acid–collagen (HA–collagen) composite hydrogel using riboflavin-mediated blue-light activation and examined how the collagen-to-HA ratio modulates physicochemical and biological properties. Storage modulus scaled with HA content (Col4HA1: 156 Pa; Col3HA1: 216 Pa; Col2HA1: 824 Pa vs. 6 Pa for Matrigel), and organoid formation efficiency was inversely correlated with stiffness: Col2HA1 yielded significantly fewer organoids than Matrigel, whereas Col4HA1 fell within a mechanically permissive range and supported organoid formation comparable to Matrigel. Col4HA1 retained 48.6 ± 2.3% of its mass throughout the 14-day organoid culture period, whereas Matrigel underwent complete degradation by Day 10. Organoids in Col4HA1 maintained stemness (Lgr5, Sox9) and differentiation markers (Villin), avoided stress-associated lysozyme elevation and preserved E-cadherin/β-catenin junctions and epithelial polarity. Critically, Col4HA1 sustained stable Lgr5 expression across passages, whereas Matrigel showed passage-dependent Lgr5 elevation indicative of hyperproliferative drift. The photo-crosslinkable HA–collagen hydrogel offers a chemically defined, tunable and reproducible Matrigel alternative for intestinal organoid research and regenerative-medicine applications.

## Graphical Abstract

**Graphical Abstract:**
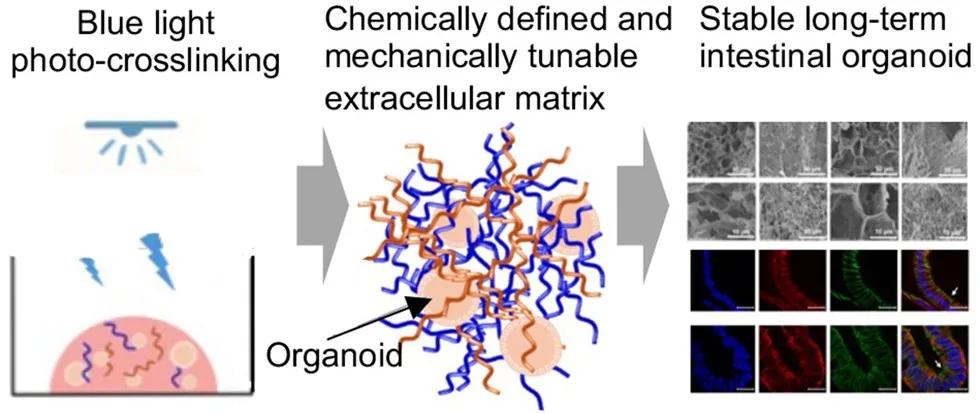

## Introduction

Organoids are advanced *in vitro* 3D culture systems that replicate the cellular architecture, spatial organization and physiological functionality of native tissues while preserving critical cell–cell and cell–matrix interactions [1]. They have emerged as powerful platforms for applications in drug screening, disease modeling and precision medicine [2–4]. The successful formation and maintenance of organoids critically depend on the extracellular matrix (ECM), which not only provides structural support but also delivers biochemical and biomechanical cues essential for cell growth, polarity and lineage specification [5].

The most widely used ECM for organoid culture is Matrigel, a basement membrane matrix derived from Engelbreth–Holm–Swarm mouse sarcoma. Its composition includes laminin (∼60%), collagen IV (∼30%), entactin (∼8%) and the heparan sulfate proteoglycan perlecan (∼2–3%), along with a spectrum of growth factors that facilitate ECM-mediated signaling and support organoid formation [6]. Despite its broad utility, Matrigel has several notable limitations. Its undefined composition makes it difficult to predict biological effects in human systems, while significant batch-to-batch variability undermines experimental reproducibility [7]. Moreover, its physical and biochemical properties cannot be precisely tuned, limiting the ability to modulate the ECM environment to direct specific cellular behaviors and achieve consistent biological outcomes [8]. These limitations underscore the need for a well-defined and tunable alternative to Matrigel for organoid culture.

Matrigel gelation is driven by temperature-dependent physical crosslinking over a broad range (22–37°C), in which entactin acts as a linker between laminin and collagen to form a hydrogel-like network [6]. This process, however, suffers from variable gelation times—ranging from minutes to hours—and offers limited control over network architecture, thereby constraining reproducibility. While several groups have explored hyaluronic acid–collagen (HA–collagen) composites as potential ECM alternatives, most prior studies have relied on chemical crosslinking methods or temperature-dependent gelation, both of which offer limited control over network formation kinetics and spatial homogeneity [9]. Moreover, existing approaches have largely focused on optimizing individual physicochemical parameters—such as stiffness or swelling—without systematically examining how compositional variations influence the biological hierarchy of stem cell maintenance, lineage differentiation and epithelial organization within organoid systems. In contrast, photo-crosslinking employs light irradiation to induce gelation, enabling precise spatiotemporal control and allowing cell encapsulation with minimal cytotoxicity. Recent advances in stem cell bioprinting have further highlighted the importance of integrating endogenous self-organization with exogenous matrix control to guide stem cell organization, tissue polarity and morphogenesis [10]. For HA-based hydrogels, photo-crosslinking is commonly achieved using methacrylated HA (MA-HA) and thiolated HA (SH-HA) in the presence of riboflavin phosphate as a photoinitiator. Upon blue-light exposure, a thiol-ene reaction occurs, resulting in stable hydrogel formation [11]. Collagen, a widely studied natural biomaterial, is well recognized for its biocompatibility, biodegradability and ability to promote cell adhesion [12]. Collagen gelation occurs via fibrillogenesis, where collagen molecules self-assemble into fibrils. In photo-crosslinkable collagen hydrogels, riboflavin phosphate activation facilitates covalent crosslinking between collagen carbonyl groups, yielding a mechanically stable network [13]. Thus, the inherent limitations of Matrigel’s temperature-dependent gelation can be effectively addressed using photo-crosslinkable HA–collagen hydrogels.

Beyond providing a physical scaffold, the ECM serves as a key regulator of cell fate by mediating cell–cell and cell–matrix interactions. In organoid cultures, the establishment of robust adherens junctions and the maintenance of stem cell self-renewal are essential for sustaining epithelial polarity and tissue architecture [14, 15]. Among the signaling pathways that integrate these processes, the Wnt/β-catenin axis plays a central role in regulating the balance between self-renewal and differentiation of intestinal crypt stem/progenitor cells, with E-cadherin and β-catenin serving as dual regulators of both adherens junction integrity and Wnt-dependent stemness signaling [16]. ECM composition directly influences the stability of this cadherin-catenin complex and the mechanical cues that govern collective epithelial morphogenesis during organoid development [17, 18].

In this study, we hypothesized that a photo-crosslinkable HA–collagen composite hydrogel with tunable collagen-to-HA ratios can recapitulate the physicochemical properties of Matrigel while providing a chemically defined and reproducible microenvironment for intestinal organoid culture. To test this, we systematically varied the collagen-to-HA ratio and characterized the resulting hydrogels in terms of mechanical properties (stiffness, viscosity, stress relaxation), structural features (pore size, fibril architecture) and biological performance including cell viability, organoid formation efficiency, stemness marker expression and epithelial polarity. We further evaluated multi-passage stability to assess whether Col4HA1 supports homeostatic stem cell maintenance superior to that of Matrigel. Collectively, this work aims to establish design principles linking matrix composition to organoid performance, positioning photo-crosslinkable HA–collagen hydrogels as a chemically controlled and mechanically versatile alternative to Matrigel for intestinal organoid systems.

## Materials and methods

### Fabrication of crosslinked hydrogel

To establish a reproducible and tunable ECM substitute for organoid culture, photo-crosslinkable HA–collagen composite hydrogels were prepared with varying collagen-to-HA ratios to modulate their mechanical properties. MA-HA and SH-HA were both purchased from Blafar (Dublin, Ireland). MA-HA and SH-HA (MW: 450 kDa; degree of methacrylation and thiol substitution: 50%, respectively, as specified by the manufacturer; Blafar, Dublin, Ireland) were dissolved in phosphate-buffered saline (PBS; Gibco, Eggenstein, Germany) to a concentration of 10 mg/mL. Then, MA-HA solution and SH-HA solution were mixed in a 1:1 (v/v) ratio. Type I atelocollagen of porcine origin (lot no. BA10061; molecular weight: 300 kDa; Dalim, Seoul, Korea) was dissolved in 0.02 M acetic acid (Samchun, South Korea) to a concentration of 10 mg/mL. Then, 1 N NaOH (Thermo Fisher Scientific, Waltham, MA, USA), 10× PBS (Thermo Fisher Scientific, Waltham, MA, USA) and DI water were used to make a 3 mg/mL collagen solution with pH 7.4. The prepared collagen was incubated at 37°C with 5% CO_2_ for 15 min before being used for organoid seeding. HA solution was mixed with the pre-incubated collagen. The collagen-to-HA ratio (Col4HA1, Col3HA1, Col2HA1, 4:1, 3:1 and 2:1 v/v ratios, respectively) was selected to cover the physiologically appropriate stiffness range of the intestinal mucosa (approx. 0.2–1 kPa) while ensuring that collagen remains the major integrin binding component [19, 20]. Low collagen ratios (Col1HA1 and HA dominance) were excluded as they showed insufficient adhesion in preliminary viability tests.

Riboflavin 5′-phosphate sodium salt hydrate (RFP; Sigma-Aldrich, Flanders, NJ, USA) was prepared at 1% (w/v) and then added to the HA–collagen hydrogel solution at a concentration of 0.005% (v/v). HA–collagen solutions were mixed with cells and seeded to encapsulate the cells within a dome structure. The hydrogel precursor solution was irradiated with blue light (385 nm, 5 mW/cm²; Altis, Incheon, South Korea) to produce a crosslinked hydrogel.

### Fourier transform infrared spectroscopy

A Fourier transform infrared spectrophotometer (FTIR) (iS50; Thermo Fisher Scientific, MA, USA) was used to investigate the chemical changes of the hydrogel crosslinked by light irradiation. The hydrogel samples of each group were prepared as described in fabrication of crosslinked hydrogel and lyophilized for 24 h. The lyophilized samples were mixed with KBr (Sigma-Aldrich, Flanders, NJ, USA), and the transmittance was measured in the range of 3000–500 cm^−1^.

### Scanning electron microscopy

The microstructural morphology of the hydrogels was examined using a scanning electron microscope (SEM; SU8600, Hitachi, Japan). For sample preparation, 200 µL of HA–collagen hydrogel solution was photo-crosslinked under blue-light irradiation (385 nm) for 1 min, while Matrigel samples were prepared by 1:1 dilution with the medium followed by thermal crosslinking at 37°C with 5% CO_2_ for 15 min. The crosslinked hydrogel was swollen in PBS for 24 h and then freeze-dried to preserve the structural integrity. The samples were then sectioned to expose their internal structures, sputter-coated with platinum for 120 s under vacuum conditions and examined by SEM to analyze the 3D structure of the hydrogel.

### Rheological characterization

Rheological measurements were performed using a rheometer (MCR92; Anton Paar, Austria) with a 25-mm parallel plate geometry at a gap height of 0.5 mm. All samples (200 µL) were measured 30 min after preparation to ensure complete crosslinking. Temperature-dependent gelation behavior was analyzed by temperature sweep measurements from 0 to 60°C, with fixed strain at 1% and frequency at 1 Hz. For photo-crosslinking analysis, samples containing 0.005% (v/v) RFP were loaded onto a plate set at 37°C onto a glass-bottomed plate at 37°C, and measurements were conducted during blue-light irradiation (1 min). Dynamic mechanical properties were characterized through oscillatory measurements with 1% strain over a frequency range of 0.1–10 Hz. Storage modulus (Gʹ) and loss modulus (G″) were recorded to evaluate the viscoelastic properties. Viscosity measurements were conducted at a constant shear rate (10 Hz), and shear-thinning behavior was assessed by varying the shear rate from 1 to 100 Hz. Stress relaxation behavior was evaluated by applying step strains of 10, 30 and 60%. Time constants were normalized to the lowest applied strain, and data were processed using a median filter (window size: 10 points) to minimize instrumental noise [21].

### Swelling and weight distribution of hydrogel

To assess the crosslinking density and long-term stability of the photo-crosslinked HA–collagen hydrogels, swelling and weight distribution analyses were performed. These parameters are directly related to the hydrogel’s ability to regulate water uptake, maintain a stable 3D microenvironment and sustain nutrient diffusion and cell-matrix interactions critical for organoid stemness. For swelling analysis, hydrogel samples were freeze-dried and weighed to obtain the dry weight (*W*_d_), then immersed in PBS at 37°C for 24 h to reach equilibrium. The swollen weight (*W*_s_) was measured, and the swelling ratio was calculated using the following formula:


$$\mathrm{Swelling}\;\mathrm{ratio}\;(\%)=\frac{\left(W_{s}-W_{d}\right)}{W_{d}}\times 100\%.$$

For stability assessment, morphological and gravimetric changes were monitored over 14 days of culture (Days 0, 4, 7, 10 and 14). Hydrogels were weighed at each time point, and values were normalized to the initial weight.

### Organoid seeding

To evaluate the biological performance of the photo-crosslinked HA–collagen hydrogels as a defined ECM substitute, primary intestinal and colonic organoids were established from C57BL/6 mice. Seven- to eight-week-old C57BL/6 mice were sacrificed to obtain intestinal crypts for organoid culture. All animal experiments were conducted in accordance with the guidelines and regulations approved by the Institutional Animal Care and Use Committee of Gachon University (GU1-2024-IA0057-00). All procedures were performed to obtain primary cells for organoid culture and were carried out in full compliance with institutional and national guidelines for the care and use of laboratory animals. The tissues were cut into 5-mm segments and incubated in 2 and 20 mM EDTA (Sigma-Aldrich, Flanders, NJ, USA) for 1 h at 4°C, respectively. Intestinal crypts were isolated following standard EDTA chelation and filtration, providing stem cell-rich fractions for culture.

The isolated crypts were then suspended in HA–collagen hydrogel or Matrigel, prepared according to experimental conditions. A total of 30 µL of the crypt-hydrogel suspension was carefully pipetted onto the center of each well in a pre-warmed culture plate, where it formed a dome-like structure due to surface tension. For crypts mixed with Matrigel, a 1:1 mixture with organoid culture medium was used prior to seeding.

Each dome was exposed to blue light for 15 s to initiate crosslinking, with each 30 µL dome containing approximately 500 crypts. After cross-linking, domes were incubated at 37°C for 30 min, after which a complete medium containing R-spondin-conditioned medium, noggin-conditioned medium, B27 (Gibco, Eggenstein, Germany), GlutaMAX™ (Gibco, Eggenstein, Germany), HEPES (Sigma-Aldrich, Flanders, NJ, USA), N2 (Thermo Fisher Scientific, Waltham, MA, USA), NALC (Sigma-Aldrich, Flanders, NJ, USA), EGF (Proteintech Group Inc., Rosemont, IL, USA) and Y-27632 (TOCRIS, UK) in adDMEM was added. Cultures were maintained for 14 days, with medium containing Y-27632 supplied for the first 2 days post-seeding and subsequently replaced with complete medium without Y-27632. Medium changes were performed every 2–3 days.

### Propidium iodide and Calcein AM staining

To determine whether the photo-crosslinked HA–collagen hydrogel preserves cell viability, dual live/dead staining was performed at Day 7. Cell viability was assessed 7 days after seeding. On Day 7 of culture, 3 µM of propidium iodide (PI; Invitrogen, Waltham, MA, USA) and 3 µM of Calcein AM (Invitrogen) were added to serum-free medium and incubated for 30 min. Following incubation, cell viability was evaluated using fluorescence microscopy and the number of stained cells was quantified using ImageJ software. Cell viability within the photo-crosslinked HA–collagen hydrogel was compared to that in Matrigel, allowing us to assess whether the defined hydrogel supports survival at levels comparable to the current standard while offering improved reproducibility.


$$\text{PI-positive}\;\text{cell}\;(\%)=\frac{\text{Number}\;\text{of}\;\text{PI–positive}\;\text{cell}}{\text{Number}\;\text{of}\;\text{Calcein}\;\text{AM–positive}\;\text{cell}+\text{Number}\;\text{of}\;\text{PI–positive}\;\text{cell}\;}\times \;100\%.$$

### Immunofluorescence analysis of organoid

To elucidate the role of the ECM in maintaining epithelial integrity and regulating Wnt/β-catenin signaling, organoids cultured for 14 days were subjected to immunofluorescence analysis. Organoids were fixed in 4% formaldehyde, embedded in paraffin and sectioned onto slides. The slides were sequentially rehydrated using xylene, followed by graded ethanol solutions (100, 95, 70 and 50%). After rehydration, sections were blocked with 3% normal goat serum for 1 h and incubated with primary antibodies overnight at 4°C. The primary antibodies included E-cadherin (Santa Cruz, 1:50), a core adherens junction protein essential for epithelial polarity, and β-catenin (Abcam, 1:50), which functions both as a structural component of the cadherin–catenin complex and as a key effector in Wnt signaling. Alexa Fluor 488 (Invitrogen) and Alexa Fluor 568 (Invitrogen) secondary antibodies were applied at a 1:200 dilution. Confocal imaging was performed using a Zeiss LSM880 microscope. Morphological parameters, including cell height and cell shape index, were quantified using ImageJ software. These parameters were chosen because they reflect epithelial polarization and structural organization, providing a functional readout of how ECM composition influences cadherin–catenin complex stability and collective morphogenesis during organoid development.


$$\mathrm{Cell}\;\mathrm{shape}\;\mathrm{index}=\frac{4\pi \times c\text{ell}\;\text{area}}{{\;(\text{cell}\;\text{perimeter})}^{2}\;}.$$

### Western blot

To investigate whether the photo-crosslinked HA–collagen hydrogel supports intestinal stemness and lineage specification, Western blot analysis was performed on organoids cultured for 14 days. Organoids cultured for 14 days were subjected to protein extraction using NP40, and the extracted proteins were separated via SDS-PAGE. Following separation, the membrane was blocked with 5% albumin and incubated with primary antibodies overnight at 4°C. The primary antibodies used were Lgr5 (Abcam, 1:10000), Sox9 (Abcam, 1:1000), Villin (Santa Cruz, 1:1000) and Lysozyme(LYZ) (Abcam, 1:10000). Secondary antibodies, including Anti-mouse IgG HRP Conjugate and Anti-rabbit IgG HRP Conjugate (Promega), were diluted at 1:7500.

### Statistical analysis

All data are presented as mean ± standard deviation (SD). Statistical significance, except for cell height and cell shape index, was assessed using one-way analysis of variance followed by Tukey’s *post hoc* test. Cell height and cell shape index were compared between two groups (Matrigel and Col4HA1) using independent *t*-tests, as only two conditions were assessed for these parameters. Differences were considered statistically significant at *P *< 0.05, *P *< 0.01 and *P *< 0.005. All biological experiments were performed in at least three independent experiments, each conducted with separate organoid isolations.

## Results

### Distinct crosslinking mechanisms define the mechanical properties of HA–collagen hydrogels

Rheological analyses revealed fundamental differences between temperature-dependent and photo-induced crosslinking systems. Matrigel and collagen gels underwent thermosensitive gelation, with the onset of storage modulus (G′) increase observed at 27 and 35°C, respectively. However, both exhibited limited final stiffness (Matrigel: 6.48 ± 0.68 Pa; collagen: 10.82 ± 1.65 Pa) (Figure 1A).

**Figure 1:**
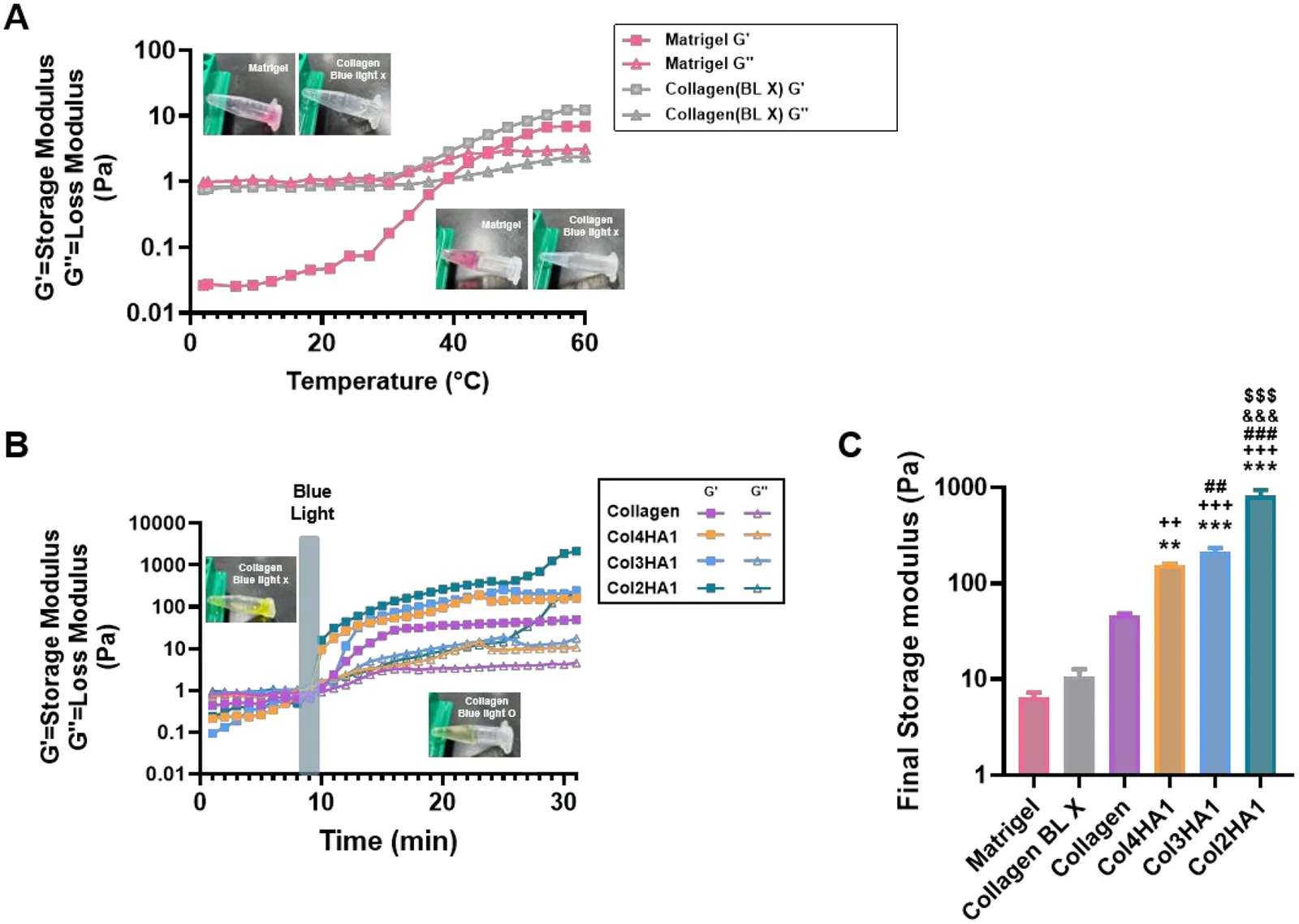
Rheological characterization of matrix crosslinking mechanism. (**A**) Temperature-dependent changes in Gʹ and Gʺ of Matrigel and Collagen without blue-light exposure (BL X). (**B**) Evolution of storage modulus during photo-crosslinking of Collagen, Col4HA1, Col3HA1, Col2HA1. (**C**) Comparison of final storage modulus values across different matrix formulations after crosslinking. The *, +, #, & and $ symbols indicate differences compared with the Matrigel, Collagen BL X, Collagen, Col4HA1, Col3HA1 group at *P *< 0.05, respectively. **, ++, ## and ***, +++, ###, &&&, $$$ indicate *P *< 0.01 and *P *< 0.005, respectively.

In contrast, photo-crosslinking systems exhibited rapid gelation kinetics upon blue-light exposure in the presence of RFP. The storage modulus increased immediately after light irradiation, indicating efficient crosslink formation (Figure 1B). Quantitative analysis of the final storage modulus values demonstrated significant differences among the various formulations: Matrigel (6.48 ±0.68 Pa), temperature-crosslinked collagen (10.82 ± 1.65 Pa), photo-crosslinked collagen (46.69 ± 1.68 Pa), Col4HA1 (155.75 ± 4.03 Pa), Col3HA1 (215.84 ± 14.53 Pa) and Col2HA1 (823.69 ± 102.31 Pa) (Figure 1B and C). As demonstrated in Figure 1C, the resulting storage moduli–Col4HA1 (∼155 Pa), Col3HA1 (∼216 Pa) and Col2HA1 (∼824 Pa)—represent near-optimal, supra-optimal and excessive stiffness conditions relative to this range, while collagen-only (∼47 Pa) and Matrigel (∼6.5 Pa) served as lower-stiffness controls. This design allowed us to establish a systematic structure-function relationship between matrix mechanics and organoid performance.

Photo-crosslinked collagen exhibited a 4.3-fold increase in storage modulus compared to temperature-crosslinked collagen. Moreover, HA addition significantly enhanced the storage modulus (G′), indicating increased stiffness and elastic stability of the hydrogel network. Based on systematic optimization of riboflavin concentration and light exposure time (Supplementary Figure S1A and B), all subsequent experiments utilized 0.005% (v/v) riboflavin with 15-s blue-light exposure for organoid seeding.

### Composite hydrogels integrate structural features of collagen fibrils and HA-derived pores

FTIR spectroscopy revealed characteristic molecular signatures of the hydrogel components (Figure 2A and B). The collagen spectrum exhibited distinctive absorption peaks at 1649 cm^−1^ (C = O, Amide I), 1541 cm^−1^ (N–H, Amide II) and 1231 cm^−1^ (C–N, Amide III). The HA spectrum showed characteristic peaks at 2922 cm^−1^ (C–H stretching), 1418 cm^−1^ (symmetric C–O stretching of carboxyl groups), 1130 cm^−1^ (C–O–C stretching) and 1045 cm^−1^ (PO43-) [22–24]. In the HA–collagen matrices (Col4HA1, Col3HA1 and Col2HA1), the Amide I peak shifted to 1640 cm^−1^, the HA carboxyl peak shifted to 1408 cm^−1^, while no additional peaks were observed.

**Figure 2:**
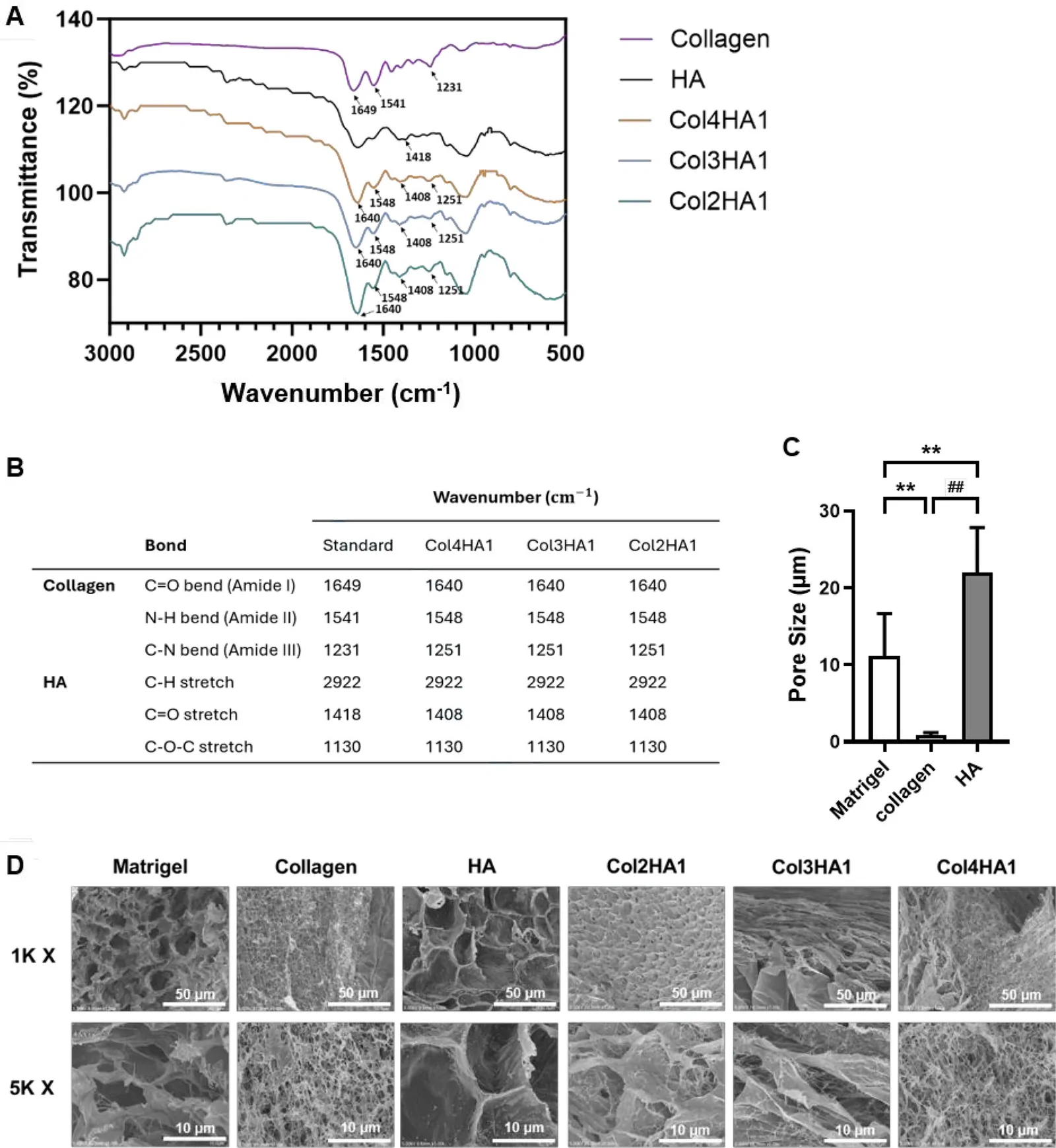
Chemical composition and structural characterization of hydrogel matrices. (**A**) FTIR spectra showing characteristic peaks of Collagen, HA and HA–collagen matrices (Col4HA1, Col3HA1, Col2HA1). (**B**) Summary of key functional groups and their corresponding wavenumbers in FTIR analysis. (**C**) Quantitative analysis of matrix pore sizes for Matrigel, Collagen and HA hydrogels (*n* = 10). (**D**) Representative SEM micrographs of hydrogel cross-sections at different magnifications showing distinct network architectures. The ** and ## symbols indicate differences compared with the Matrigel and Collagen group at *P *< 0.01.

Although rheological analysis clearly demonstrated the successful gelation of the HA–collagen matrices upon photo-crosslinking, the FTIR spectra did not exhibit distinct new absorption bands corresponding to the thiol-ene or methacrylate crosslinking reaction. This absence of detectable chemical changes is likely due to the relatively low degree of functional group conversion and the dominance of strong amide and saccharide vibrational bands, which can mask subtle variations in the spectra. Therefore, the FTIR results indicate that the primary molecular structures of collagen and HA were preserved after crosslinking, without the appearance of degradation-related peaks, and serve as supportive evidence rather than direct proof of crosslink formation.

Regarding the 1700–2500 cm^−1^ region: neither collagen nor HA exhibits characteristic diagnostic absorptions in this range. The weak, broad features visible in this region correspond to an amide overtone/combination band from collagen (∼1800–2000 cm^−1^) and low-intensity baseline contributions associated with the KBr pellet preparation. Importantly, no new absorption bands indicative of crosslink formation were identified in this region, consistent with our interpretation that FTIR serves as supportive evidence of structural preservation rather than direct proof of covalent crosslink formation.

SEM analysis demonstrated distinct network architectures among the different hydrogel formulations (Figure 2D). Quantitative analysis of pore sizes revealed significant differences: Matrigel (11.15 ± 5.23 µm), collagen (0.84 ± 0.36 µm) and HA (23.64 ± 5.72 µm) (Figure 2C). The HA–collagen matrices exhibited a hierarchical structure where collagen fibrils were integrated within the larger HA-derived pores. Increasing HA content correlated with larger pore sizes, whereas collagen enrichment reinforced fibrillar features.

### HA–collagen hydrogels demonstrate enhanced stability and tunable swelling

Long-term stability analysis revealed distinct degradation patterns among the different matrices during the 14-day culture period (Figure 3A). Matrigel and collagen showed complete structural deterioration by Days 10 and 7, respectively. In contrast, all HA–collagen matrices maintained their structural integrity throughout the culture period. Quantitative weight loss measurements confirmed these observations, with Matrigel and collagen showing complete degradation (0% remaining mass) by Days 7 and 10, respectively (Figure 3B). The HA–collagen matrices exhibited superior stability, retaining significant portions of their initial mass after 14 days: Col4HA1 (48.64 ± 2.27%), Col3HA1 (52.61 ± 4.73%) and Col2HA1 (69.65 ± 8.34%).

**Figure 3:**
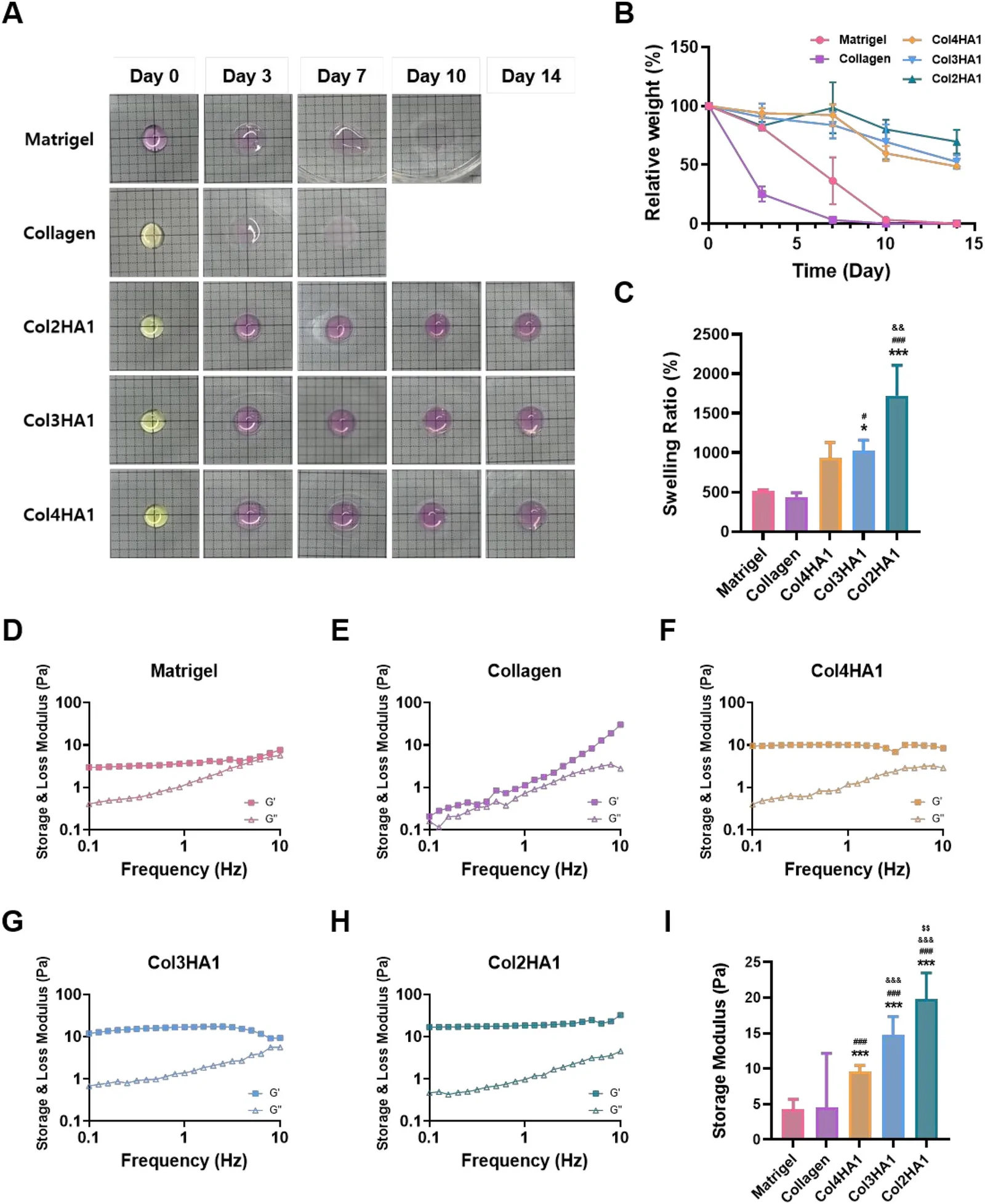
Physicochemical stability and mechanical analysis of hydrogel matrices. (**A**) Representative images showing structural stability during 14-day culture period. (**B**) Quantitative analysis of matrix degradation expressed as percentage of remaining weight over time. (**C**) Equilibrium swelling ratios of different matrix formulations after 24 h PBS immersion. (**D**–**H**) Frequency-dependent changes in Gʹ and Gʺ moduli for: (**D**) Matrigel, (**E**) Collagen, (**F**) Col4HA1, (**G**) Col3HA1 and (**H**) Col2HA1. (**I**) Comparison of final storage modulus values across different matrix formulations measured at 1 Hz. The *, #, & and $ symbols indicate differences compared with the Matrigel, Collagen, Col4HA1 and Col3HA1 group at *P *< 0.05, respectively. **, &&, $$ and ***, ###, &&& indicate *P *< 0.01 and *P *< 0.005, respectively. Values represent mean ± SD (*n* = 3).

Swelling behavior analysis demonstrated varying degrees of water absorption among the matrices (Figure 3C). The measured swelling ratios were as follows: Matrigel (520.33 ± 8.18%), collagen (441.43 ± 41.45%), Col4HA1 (935.98 ± 158.05%), Col3HA1 (1026 ± 109.12%) and Col2HA1 (1772.06 ± 315.25%). The swelling capacity increased proportionally with HA content in the HA–collagen matrices.

Rheological characterization revealed distinct viscoelastic properties among the matrices (Figure 3D–I). Frequency sweep measurements showed that in Matrigel, Gʺ exceeded Gʹ at higher frequencies (Figure 3D), while collagen exhibited increasing Gʹ with frequency (Figure 3E). The HA–collagen matrices maintained constant Gʹ values higher than Gʺ across all frequencies (Figure 3F–H). The final storage modulus values were as follows: Matrigel (4.24 ± 1.41 Pa), collagen (4.56 ± 7.42 Pa), Col2HA1 (19.78 ± 3.60 Pa), Col3HA1 (14.82 ± 2.47 Pa) and Col4HA1 (9.62 ± 0.81 Pa) (Figure 3I).

Viscosity measurements revealed significant differences among the matrices. The measured viscosity values were as follows: Matrigel (1.06 ± 0.31 mPa·s), collagen (0.11 ± 0.06 mPa·s), Col2HA1 (105.19 ± 5.82 mPa·s), Col3HA1 (53.42 ± 4.86 mPa·s) and Col4HA1 (13.00 ± 3.01 mPa·s) (Figure 4A). Under varying shear rates, all matrices demonstrated shear-thinning behavior with viscosity decreasing at higher shear rates (Figure 4C). The HA–collagen matrices showed a positive correlation between HA content and viscosity, with Col2HA1 exhibiting the highest viscosity and the most gradual decrease in viscosity with increasing shear rate.

**Figure 4:**
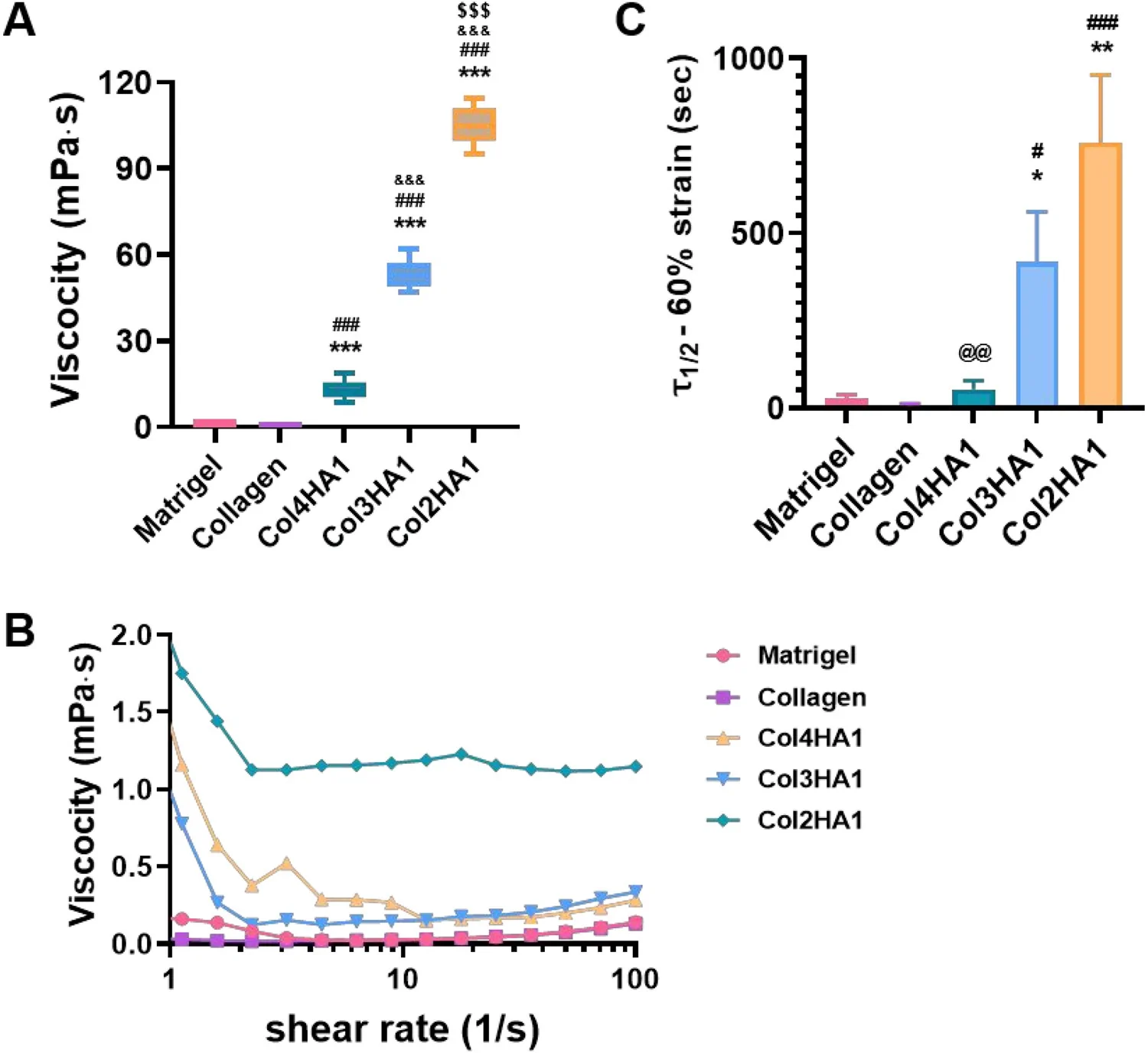
Viscoelastic behavior analysis of hydrogel matrices. (**A**) Steady-state viscosity values of different matrix formulations measured at 37°C. (**B**) Shear rate-dependent viscosity profiles demonstrating shear-thinning behavior. (**C**) Comparison of τ1/2 relaxation times at 60% strain across different matrix formulations. The *, #, &, $ and @ symbols indicate differences compared with the Matrigel, Collagen, Col4HA1, Col3HA1, Col2HA1 group at *P *< 0.05, respectively. **, &&, @@ and ***, ###, &&&, $$$ indicate *P *< 0.01 and *P *< 0.005, respectively. Values represent mean ± SD (*n* = 3).

Stress relaxation analysis demonstrated strain-dependent viscoelastic behavior across all matrices (Supplementary Figure S2). At 60% strain, the measured τ1/2 relaxation times were as follows: Matrigel (27.10 ± 7.04 s), collagen (6.27 ± 4.12 s), Col2HA1 (759.35 ± 136.95 s), Col3HA1 (418.66 ± 100.67 s) and Col4HA1 (52.98 ± 17.70 s) (Figure 4B). The HA–collagen matrices exhibited significantly longer relaxation times compared to both Matrigel and collagen, with relaxation time increasing proportionally with HA content.

### HA–collagen hydrogels maintain cell viability and support organoid formation

Cell viability was assessed after 7 days of culture using a live/dead assay with PI and Calcein AM staining (Figure 5A). Quantitative analysis revealed that the collagen-only group exhibited approximately 3-fold higher PI-positive cells compared to other hydrogel conditions, indicating significant cell death. In contrast, the HA–collagen composite hydrogels demonstrated cell viability comparable to that observed in Matrigel cultures, independent of the HA concentration (Figure 5C).

**Figure 5:**
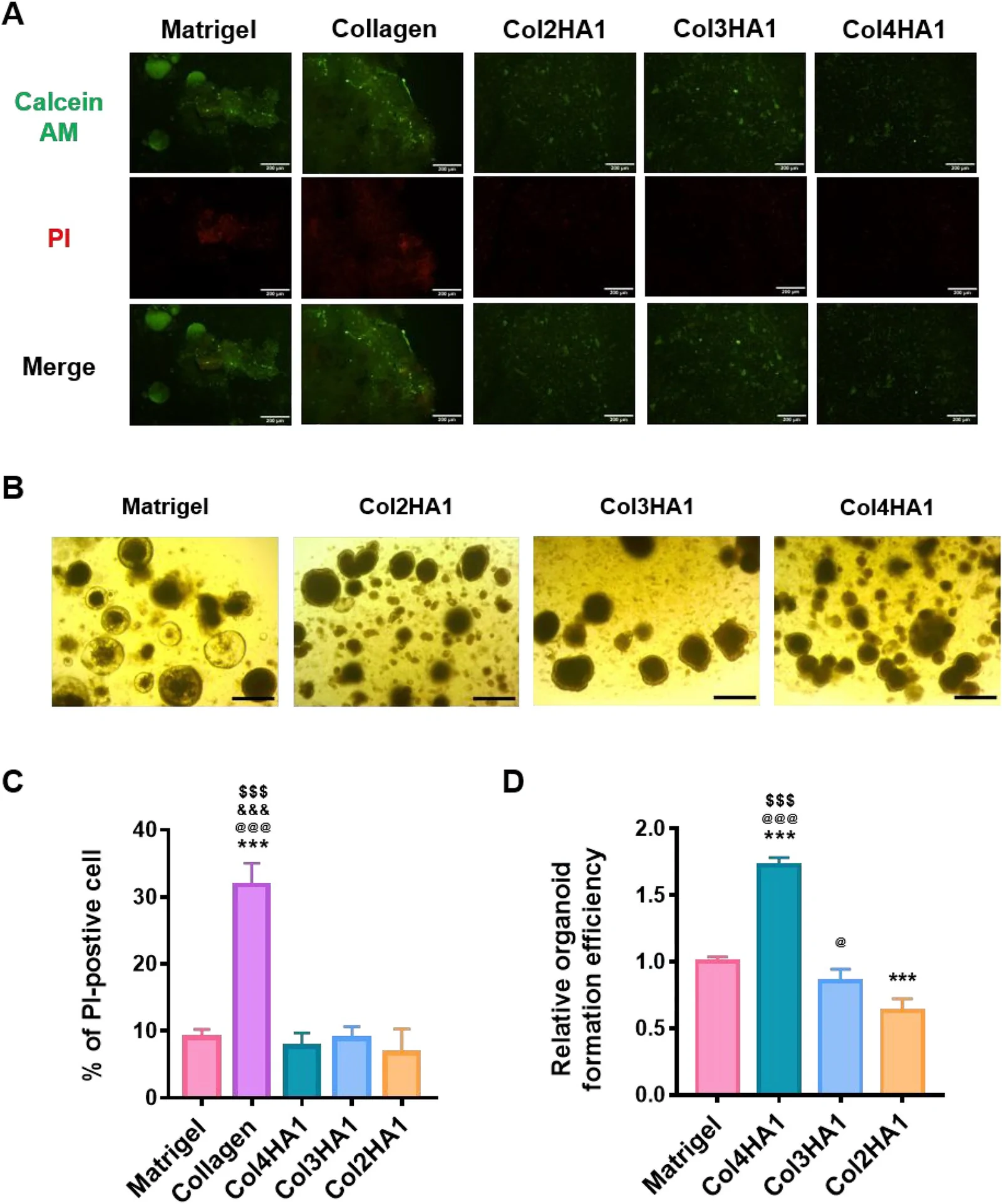
Cell viability and organoid formation efficiency in hydrogel. (**A**) Calcein AM and PI staining of cells cultured for 7 days in Matrigel, collagen, Col2HA1, Col3HA1 and Col4HA1. Scale bar = 200 µm. (**B**) Intestinal organoid formation cultured in Matrigel, Col2HA1, Col3HA1 and Col4HA1 for 14 days. Scale bar = 400 µm. (**C**) Percentage of PI-stained cells relative to the total number of cells cultured on each gel. (**D**) Relative organoid formation capacity in each gel compared to Matrigel. Bars represent mean ± S.D. (*n* = 3). The *, @, $ and & symbols indicate differences compared with the Matrigel, Col2HA1, Col3HA1 and Col4HA1 group at *P *< 0.05, respectively. ***, @@@, $$$, &&& indicate *P *< 0.005.

To identify the optimal hydrogel composition that supports intestinal organoid development, we systematically investigated organoid formation using hydrogels with varying collagen-to-HA ratios (Figure 5B). Following a 14-day culture period, organoid formation efficiency was quantified in all experimental groups, excluding the collagen-only condition due to compromised cell viability (Figure 5D). The Col2HA1 hydrogel composition yielded significantly fewer organoids compared to Matrigel controls. However, increasing the collagen content in Col3HA1 and Col4HA1 formulations resulted in significantly enhanced organoid formation relative to Col2HA1. Notably, we observed a positive correlation between collagen concentration and organoid formation efficiency, with higher collagen ratios promoting increased organoid development. Biological validation confirmed that the selected photo-crosslinking conditions preserved Sox9 expression at levels comparable to Matrigel, ensuring that mechanical optimization did not compromise stem/progenitor cell populations (Supplementary Figure S1C and D).

### HA–collagen hydrogels support organoid intestinal differentiation and epithelial polarity

Microscopic examination revealed distinct budding structures—a defining morphological feature of intestinal organoids that reflects active stem cell compartments—across different matrix conditions (Figure 6A). Organoids in Col4HA1 exhibited well-defined budding morphologies comparable to Matrigel controls, with multiple protrusions extending from the organoid core (Figure 6A). In contrast, Col3HA1 organoids displayed fewer and smaller budding structures, suggesting compromised stem cell niche function. To validate these morphological observations at the molecular level, we performed Western blot analysis of key intestinal markers (Figure 6B). The assessment included the intestinal stem cell marker Lgr5, crypt base transcription factor Sox9 and epithelial differentiation marker Villin. Additionally, we examined lysozyme expression as an indicator of cellular stress response (Figure 6C–F). Quantitative analysis revealed that organoids cultured in Col3HA1 hydrogels exhibited significantly reduced expression of Lgr5, Sox9 and Villin compared to Matrigel, consistent with their diminished budding morphology (Figure 6A). Furthermore, Col3HA1 cultures demonstrated significantly elevated lysozyme secretion, suggesting suboptimal culture conditions and potential Paneth cell dysfunction. In contrast, organoids cultured in Col4HA1 hydrogels maintained protein expression levels comparable to Matrigel for all assessed markers, with no significant differences observed in lysozyme expression. This structure–function correlation—where robust budding morphology in Col4HA1 aligns with preserved stemness marker expression—demonstrates that the optimized mechanical properties of Col4HA1 support both the architectural and molecular hallmarks of functional stem cell niches.

**Figure 6:**
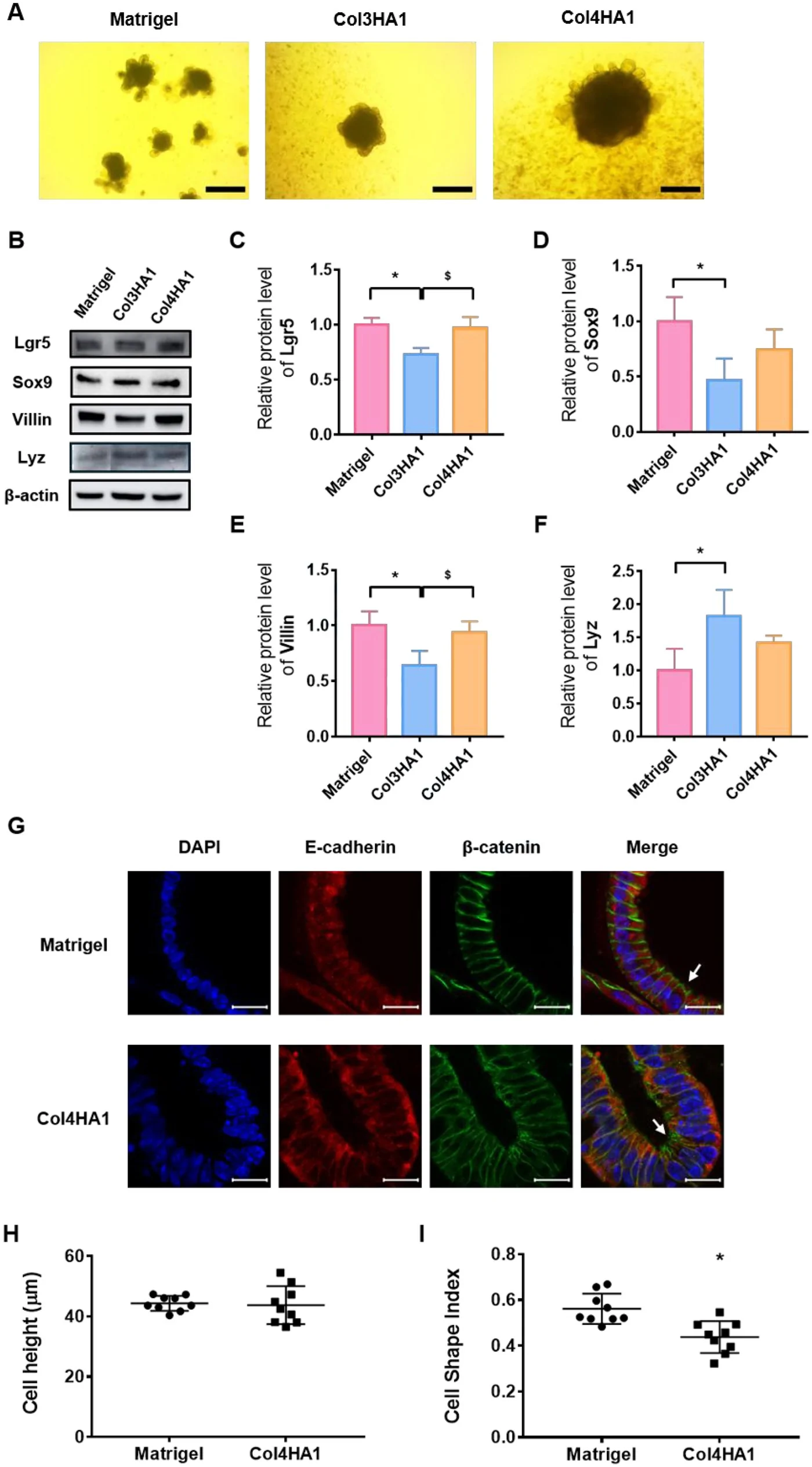
Morphological and molecular characterization of intestinal organoid differentiation in HA–collagen hydrogels. (**A**) Representative brightfield images showing budding structures. Scale bar = 200 µm. (**B**) Western blotting of intestinal organoid cultured in Matrigel, Col3HA1, Col4HA1. (**C**–**F**) Relative protein level of Lgr5, Sox9, Villin and LYZ. (**G**) Confocal image of intestinal organoid stained DAPI, E-cadherin and β-catenin. (**H**) Cell height and (**I**) cell shape index of intestinal organoid cultured in Matrigel and Col4HA1. Scale bar = 20 µm. Bars represent mean ± SD (*n* = 3). Data points represent individual measurements with horizontal lines and error bars indicating mean ± SD (*n* = 9). The * and $ symbols indicate differences compared with the Matrigel and Col3HA1 group at *P *< 0.05, respectively.

Beyond stem cell compartmentalization, the cadherin–catenin complex plays a crucial role in maintaining epithelial cell–cell adhesion and tissue organization. Immunofluorescence analysis revealed that organoids cultured in Col4HA1 hydrogels displayed cellular morphology and organization similar to those in Matrigel, with distinct co-localization of E-cadherin and β-catenin at cell–cell junctions (Figure 6G, white arrows). Complementary α-tubulin staining demonstrated that both Col3HA1 and Col4HA1 organoids maintained well-organized radial microtubule networks from apical to basal surfaces, indicating preserved cytoskeletal architecture and epithelial polarity (Supplementary Figure S3). Notably, Col3HA1 organoids exhibited organized microtubule structure despite reduced stemness marker expression, suggesting that while stem cell populations were compromised, differentiated epithelial cells maintained proper structural organization. This co-localization pattern suggests proper formation and maintenance of the cadherin-catenin complex, which is essential for epithelial barrier function. Morphometric analysis of the immunofluorescence images revealed comparable cell heights between Col4HA1 and Matrigel cultures (Figure 6H). However, the cell shape index was significantly lower in Col4HA1 compared to Matrigel, indicating altered cellular geometry while maintaining epithelial organization (Figure 6I). These findings indicate that despite the mechanical differences with Matrigel and hydrogel, Col4HA1 supported comparable organoid formation efficiency (Figure 5D), stemness marker expression (Figure 6C and D) and epithelial polarity (Figure 6G–I), suggesting that the intermediate mechanical window provided by Col4HA1 falls within the physiologically permissive range for intestinal organoid culture. Importantly, the optimized Col4HA1 was favorable with respect to both stiffness and integrin ligand presentation. Its storage modulus (∼156 Pa) lies within the physiological stiffness range of the intestinal mucosa (0.2–1 kPa) rather than being supraphysiological [19, 20]. In addition, the stiffer Col2HA1 (∼824 Pa) reduced organoid formation (Figure 5D), whereas the robust swelling and budding of Col4HA1 organoids (Figure 6A) confirm that the required 3D spatial expansion and mechanotransductive signaling were preserved. The 23.64-µm pore size, in turn, refers only to the HA-only control: in the working composite, collagen fibrils—the major integrin-binding component—are embedded within the HA-derived pores (Figure 2D), so ligand presentation is governed by the collagen network rather than by HA pore size, with the collagen-dominant Col4HA1 providing the highest ligand density among the tested composites.

### HA–collagen hydrogels maintain intestinal characteristics during passages

To assess the capacity of HA–collagen hydrogels to maintain intestinal characteristics through subsequent passages, Western blot analysis was performed to explore the expression levels of key markers before (p0) and after (p1) passage (Figure 7A and B).

**Figure 7:**
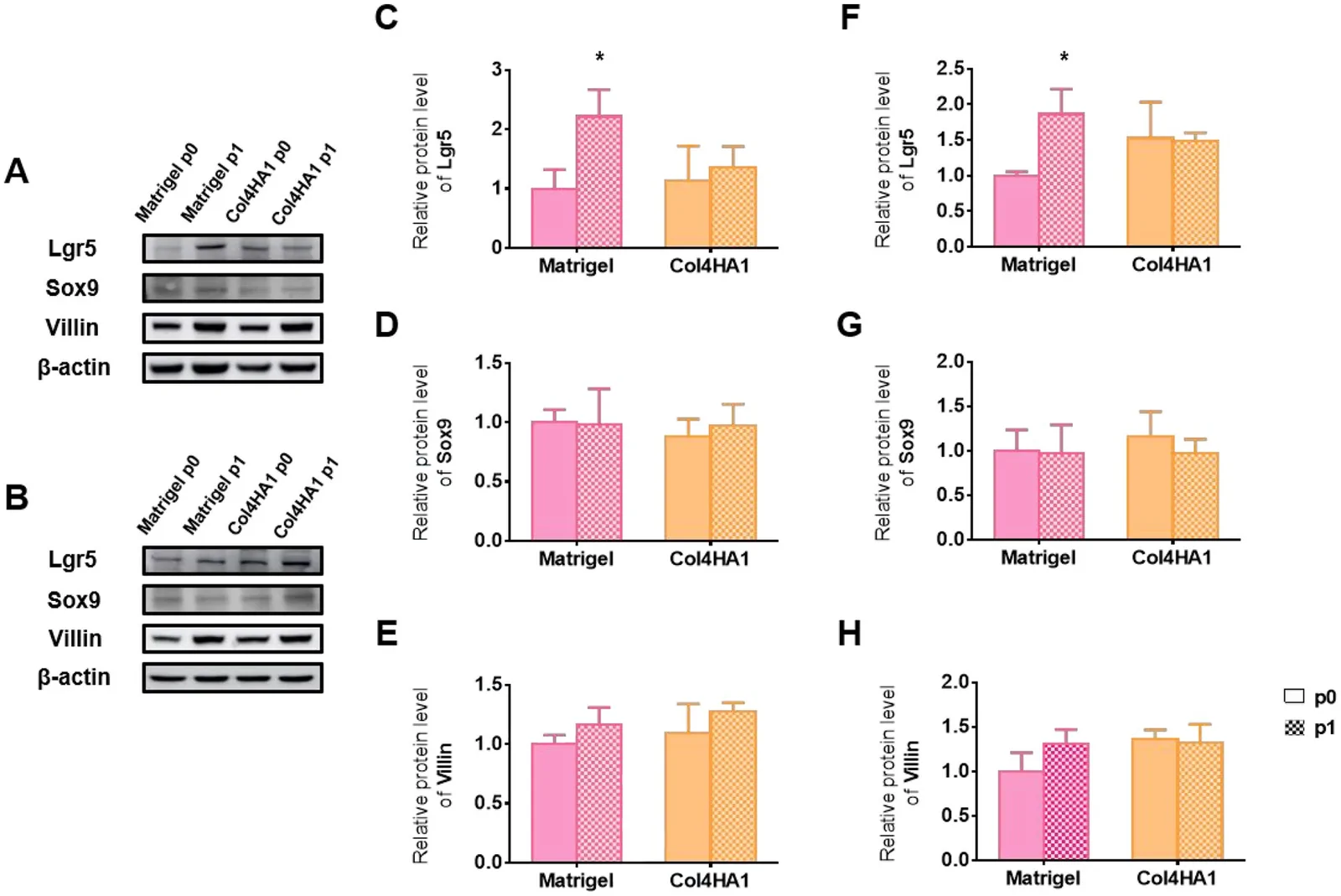
Confirmation of intestinal organoid differentiation after passage. (**A**) Western blotting images of intestinal organoid passage 0 and passage 1. (**B**) Western blotting images of colon organoid passage 0 and passage 1. (**C**–**E**) Relative protein levels of Lgr5, Sox9 and Villin of intestinal organoid. (**F**–**H**) Relative protein level of Lgr5, Sox9 and Villin of colon organoid. Bars represent mean ± SD (*n* = 3). The * symbols indicate differences compared with the Matrigel at *P *< 0.05.

In Matrigel cultures, both small intestinal and colonic organoids exhibited significantly increased expression of the stem cell marker Lgr5 following passage (Figures 7C and F). Conversely, organoids maintained in Col4HA1 hydrogels demonstrated consistent Lgr5 expression levels between p0 and p1, comparable to the expression observed in p0 Matrigel cultures. This stability in stem cell marker expression indicates that Col4HA1 hydrogels provide a more homeostatic environment for maintaining stem cell populations through passages.

The expression of Sox9, a transcription factor associated with intestinal progenitor cells (Figures 7D and G) and Villin, an epithelial differentiation marker (Figure 7E and H), remained stable across passages in both Matrigel and Col4HA1 hydrogel. The consistent expression of these markers suggests that HA–collagen hydrogel systems effectively maintain the cellular hierarchy and differentiation capacity of intestinal organoids through passages.

These findings demonstrate that Col4HA1 hydrogels support the maintenance of intestinal characteristics through subsequent passages, as evidenced by the stable expression of key intestinal markers. Moreover, the Col4HA1 system appears to provide more consistent stem cell maintenance compared to Matrigel while equally supporting the expression of differentiation markers.

## Discussion

In this study, we developed and characterized photo-crosslinkable HA–collagen hydrogels as a defined ECM substitute for intestinal organoid culture. Collagen and HA, as principal components of the natural ECM, provide excellent biocompatibility and create a microenvironment that closely mimics *in vivo* conditions [25]. HA, a highly hydrated glycosaminoglycan, plays critical roles in cellular processes including mobility, angiogenesis and signaling pathways [26]. The integration of these components enables the development of hydrogels that replicate both structural and functional properties of the ECM, with tunable mechanical properties through chemical crosslinking [27]. Unlike previous HA–collagen formulations that relied on chemical crosslinking or thermal gelation with limited control over network formation, our photo-crosslinkable system enables precise modulation of crosslink density through light dose and exposure time. This spatiotemporal control is particularly advantageous for organoid encapsulation, as it minimizes mechanical stress during cell seeding and allows for uniform matrix formation. Moreover, while earlier studies focused primarily on optimizing mechanical properties, our work systematically examines the biological consequences of matrix composition on stem cell maintenance, differentiation balance and epithelial architecture through multi-passage analysis. This composite hydrogel system, particularly Col4HA1, demonstrates enhanced mechanical stability and reproducibility while supporting organoid growth and differentiation comparable to conventional matrices.

Rheological analysis revealed that photo-crosslinking markedly increased the storage modulus (G′), indicating enhanced stiffness and elastic stability compared with thermally crosslinked collagen and Matrigel. Matrigel exhibited temperature-dependent changes in both storage and loss moduli, consistent with its thermosensitive gelation behavior (Figure 1A). In contrast, the HA–collagen hydrogel demonstrated significant alterations in rheological properties following photo-crosslinking, with measurable increases in both storage and loss moduli upon light irradiation (Figure 1B). This light-mediated gelation mechanism provides a significant advantage in terms of spatiotemporal control over the gelation process compared to the temperature-dependent Matrigel system. The incorporation of HA further amplified this effect in a ratio-dependent manner, enabling fine-tuning of matrix stiffness across a physiologically relevant range. Spectroscopic characterization of the HA–collagen composite hydrogel confirmed the preservation of the characteristic molecular signatures of both constituent biopolymers (Figure 2A and B). The composite formulation exhibited the distinctive spectral peaks associated with both collagen and HA. Consistent with this, EDC/NHS-crosslinked collagen networks similarly retain their characteristic amide signatures with minimal spectral change, because the newly formed amide crosslinks are largely masked by the native amide bands [28]. These results indicate that FTIR reports the integrity of the bulk polymer constituents rather than the small population of new crosslinks, which are masked by strong amide and saccharide bands. The covalent network is instead demonstrated by the light-triggered increase in storage modulus and by the resistance to degradation over 14 days, neither of which would occur in a non-crosslinked polymer mixture [29].

The integration of HA into collagen matrices fundamentally altered the network architecture, as demonstrated by SEM analysis. HA–collagen hydrogels exhibited hierarchical porous structures in which collagen fibrils were interwoven within HA-derived pores, thereby combining adhesive support with effective pathways for nutrient and metabolite diffusion [9, 30]. This organization contrasts with the heterogeneous composition of Matrigel and the overly compact structure of pure collagen gels, offering an optimized balance between porosity and fibrillar integrity. Such hierarchical organization is increasingly recognized as a key determinant of hydrogel functionality. Our findings are consistent with these insights, as the Col3HA1 and Col4HA1 hydrogels demonstrated structural features that supported sustained organoid viability and differentiation while avoiding the premature collapse observed in Matrigel and collagen-only conditions.

HA–collagen hydrogels exhibited significantly greater resistance to degradation than Matrigel or pure collagen, maintaining structural integrity throughout 14 days of culture. Swelling analysis showed that increased HA content enhanced water uptake, which was associated with prolonged stress relaxation times and elevated viscosity. These viscoelastic properties are known to regulate cell spreading, proliferation and differentiation in ECM-like hydrogels for 3D culture environments [31]. Such behavior contrasts sharply with the rapid instability observed in Matrigel and collagen-only gels. In addition, natural polymer-based hydrogels with tunable viscoelastic characteristics and controlled degradation profiles have been reported to provide a more consistent ECM environment for organoid and microphysiological culture systems [5]. The mechanical characterization of HA–collagen hydrogels also revealed similarities to Matrigel, while demonstrating that the incorporation of HA into collagen markedly enhanced structural stability and mechanical performance, as evidenced by rheological analyses. The temporal stability assessment of photo-crosslinked HA–collagen hydrogels in comparison with Matrigel indicated that the composite matrices can preserve a defined and consistent ECM environment throughout the culture period (Figures 3 and 5). Collectively, these findings highlight the importance of combining HA-mediated hydration with collagen-mediated fibrillogenesis to achieve both mechanical resilience and biological compatibility in hydrogel design. Critically, the mechanical profile of Col4HA1, characterized by a storage modulus of ∼156 Pa, a stress relaxation half-time of ∼53 s at 60% strain and an intermediate viscosity of ∼13 mPa·s, defines a permissive mechanical window for intestinal organoid culture. Within this window, the intermediate stiffness is consistent with integrin-mediated adhesion and cytoskeletal tension sufficient for crypt morphogenesis, while the faster stress relaxation relative to Col3HA1 (∼419 s) and Col2HA1 (∼759 s) correlates with the improved viability and budding observed in these formulations.

Collagen-only gels exhibited higher rates of cell death compared to HA–collagen composite gels (Figure 5A and C). This differential cellular viability highlights the critical role of HA in creating a permissive microenvironment for organoid survival. While collagen provides essential adhesive ligands through integrin binding, its overly compact network architecture (pore size: 0.84 ± 0.36 µm) combined with low swelling capacity (441.43 ± 41.45%, Figure 3C) creates a restrictive environment that impedes nutrient transport and waste removal. Previous work has shown that collagen networks display pronounced nonlinear viscoelasticity and strain-dependent stress relaxation, which can impose dynamic mechanical cues on embedded cells [21]. In contrast, the incorporation of HA increases water uptake, swelling capacity and porosity of collagen-based hydrogels, thereby facilitating nutrient and cytokine diffusion while modulating the overall stiffness and viscoelastic behavior [9]. Furthermore, the absence of HA eliminates key biochemical cues that regulate stem cell self-renewal through CD44-mediated Wnt/β-catenin signaling [32, 33]. This dual limitation—both mechanical restriction and biochemical insufficiency—underscores the necessity of HA incorporation for sustaining organoid viability. When evaluating organoid formation rates in HA–collagen composite gels, it is observed that fewer organoids formed in the Col2HA1 gel compared to Matrigel, while organoid formation increased with higher collagen ratios (Figure 5B and D). This systematic increase in organoid formation efficiency with higher collagen content demonstrates the importance of matrix mechanical properties in supporting organoid development. Our analysis revealed that Col4HA1 hydrogels effectively support intestinal organoid differentiation, maintaining expression levels of key markers comparable to Matrigel (Figure 6B–F).

Sox9, a crypt transcription factor expressed in the small intestinal epithelial stem cell region, regulates both stem cell proliferation and differentiation [34]. The stable expression of Sox9 and Villin, an epithelial differentiation marker, in Col4HA1-grown organoids indicates that this formulation provides a microenvironment capable of preserving the epithelial hierarchy and enabling proper lineage specification. Villin expression, in particular, reflects well-maintained absorptive epithelial identity, suggesting that Col4HA1 supports not only stem cell activity but also downstream differentiation programs essential for intestinal physiology. The presence of lysozyme-expressing Paneth cells, confirmed by lysozyme levels in the Western blot analysis, further validates the establishment of a functional stem cell niche. Paneth cells secrete essential niche factors including Wnt3, EGF and Notch ligands, which are required for sustaining the Lgr5 stem cell population [35]. Thus, the preservation of Paneth-cell-related markers in Col4HA1 suggests that the hydrogel successfully recapitulates the biochemical signaling landscape of the crypt.

The formation and maintenance of the cadherin–catenin complex in Col4HA1-grown organoids further support its suitability for organoid culture. The E-cadherin/β-catenin complex is essential for maintaining epithelial integrity and mediating the balance between barrier formation and proliferative signaling. β-catenin functions as both a structural component of adherens junctions and a co-activator of Wnt target genes, linking mechanical organization to transcriptional control [36, 37]. The preserved co-localization of E-cadherin and β-catenin in Col4HA1 organoids demonstrates that the composite hydrogel enables the stable formation of cell-cell contacts and maintains proper epithelial polarity (Figure 6G). Cytoskeletal analysis further demonstrated the superiority of the defined HA–collagen matrix: while Matrigel-grown organoids exhibited less organized microtubule architecture, Col4HA1 organoids displayed well-defined radial α-tubulin networks characteristic of properly polarized intestinal epithelium (Supplementary Figure S3). Together, these findings indicate that the defined ECM environment of HA–collagen not only maintains junctional integrity but also provides superior structural support for epithelial organization.

While the decreased cell shape index in Col4HA1 hydrogels indicates altered cellular geometry, this observation aligns with established relationships between matrix stiffness and cellular adaptation [38, 39]. Hunt *et al*. [38] demonstrated that human intestinal organoids remodel their epithelial architecture in response to engineered hydrogel stiffness, modifying polarity and junctional organization to maintain tissue-level integrity. Similarly, Caliari *et al*. [39] reported that cell spreading is highly sensitive to the viscoelastic properties of 3D hydrogels. Consistent with these findings, the lower cell shape index observed in Col4HA1 likely reflects an adaptive response to the composite matrix’s slower stress relaxation and higher fibrillar density [40]. Importantly, this geometric adaptation did not impair epithelial polarity or differentiation marker expression.

A particularly significant finding was the differential regulation of Lgr5 expression during passaging. While Matrigel cultures showed increased Lgr5 expression post-passage, Col4HA1 hydrogels maintained consistent expression levels (Figure 7C and F). This stability is crucial given that Lgr5 overexpression has been associated with colorectal cancer development and poor prognosis [41]. The maintenance of stable Lgr5, Sox9 and Villin expression suggests that Col4HA1 hydrogels may more closely mimic physiological stem cell dynamics, thereby allowing long-term organoid culture without inducing hyperproliferative drift [42]. These properties would be particularly valuable in disease modeling and drug testing applications, as matrix-induced artifacts in stem cell behavior can confound experimental interpretation.

Beyond biological compatibility, the light-mediated crosslinking strategy provides a major advantage by enabling precise control over the physicochemical characteristics of the HA–collagen matrix. Riboflavin-initiated photo-crosslinking rapidly and uniformly forms a polymer network under blue-light exposure, allowing fine modulation of crosslink density and stiffness through adjustments in light dose and exposure time—features not achievable in thermally gelled matrices such as Matrigel, which suffer from batch variability and limited structural control [11, 13]. First, the collagen:HA ratio was varied at three levels (2:1, 3:1, 4:1) at a fixed total polymer concentration. Second, riboflavin concentration and light exposure time were independently optimized against both mechanical (Gʹ) and biological (Sox9 expression) endpoints, as presented in Supplementary Figure S1. Our systematic optimization revealed that while higher riboflavin concentrations enhanced mechanical crosslinking, they concurrently reduced stem cell marker expression, highlighting the importance of balancing matrix properties with biological compatibility (Supplementary Figure S1). Prior studies have shown that blue light-induced collagen crosslinking enhances fibrillogenesis and mechanical stability [11, 13], consistent with our rheological observations demonstrating that the Col4HA1 formulation provides an optimized mechanical window that supports epithelial polarity, niche signaling and long-term organoid growth without over-stiffening. Although further investigation is needed to clarify how matrix mechanics, particularly viscoelasticity, stress relaxation and degradation behavior influence pathways such as Wnt and Notch signaling during stem cell regulation [16, 37, 43], our results indicate that photo-crosslinked HA–collagen hydrogels offer superior reproducibility and microenvironmental control. This tunability positions Col4HA1 as a promising and clinically relevant alternative to Matrigel for organoid research, disease modeling and regenerative medicine.

While photo-crosslinkable HA–collagen hydrogels supported organoid stemness and epithelial integrity comparably to Matrigel, several limitations remained. Regarding physicochemical characterization, although rheological data confirm successful network formation, direct spectroscopic verification of crosslink bonds remains incomplete; solution-state ¹H Nuclear Magnetic Resonance Spectroscopy (NMR) or X-ray Photoelectron Spectroscopy (XPS) would require specimen conditions incompatible with our swollen composite system and are beyond the current scope, and rheokinetic analysis during crosslinking would be a valuable future addition. Biologically, although key stemness and differentiation markers were stably maintained through passage (Figure 7), extended multi-passaging studies assessing long-term genetic stability over prolonged culture would further support applications such as organoid biobanking and patient-derived models for personalized medicine. Several translational considerations also remain. First, MA-HA and SH-HA synthesis involves multi-step procedures, but these are well-established and amenable to standardization, making clinical-grade scale-up an achievable goal with further process optimization [44]. Second, photo-crosslinking was optimized in this study for small-scale dome culture; the spatiotemporal control inherent to light-mediated gelation provides a practical basis for adaptation to larger formats, and evaluating crosslink uniformity across multi-well configurations will further strengthen reproducibility [45]. Third, although HA derivatives are currently more costly than collagen, this difference may diminish with scaled production and the defined composition and reproducibility of the HA–collagen system could offset the added cost in applications where experimental consistency is critical [46, 47].

Despite these limitations, our systematic characterization of mechanical–biological relationships in tunable HA–collagen matrices provides a foundation for rational ECM design in organoid culture. By allowing matrix stiffness, stress relaxation and degradation kinetics to be varied independently—unlike Matrigel’s undefined, fixed composition—the photo-crosslinking approach uniquely enables future dissection of specific mechanotransduction pathways. Such insights could guide organ-specific ECM formulations for liver, lung, pancreatic and other epithelial organoid systems, whose mechanical properties and biochemical cues differ from those of the intestine. The defined composition and reproducible manufacturing of these hydrogels further position them as candidates for clinical translation, where regulatory approval demands well-characterized biomaterials. Incorporating patient-derived organoids in Col4HA1 matrices could then validate their utility for precision medicine, including drug screening, toxicity testing and disease modeling across genetically diverse populations.

## Conclusion

The spatiotemporal control of photo-crosslinking offers further advantages over heat- or enzyme-gelled matrices. Because gelation is triggered by light on demand, the matrix can be patterned into defined geometries with locally controlled mechanical or compositional gradients—a feature difficult to achieve with conventional self-assembling ECM analogs. This makes the HA–collagen system well suited to microfluidic organ-on-a-chip platforms, where precise *in situ* gelation within confined microchannels and spatially defined cellular microenvironments are essential for reproducing tissue-level structures and physiological gradients [48]. Together, these capabilities make photo-crosslinkable HA–collagen hydrogel a versatile matrix that bridges basic organoid culture with high-throughput drug screening and biomanufacturing-oriented clinical and industrial applications.

## Supplementary Material

rbag171_Supplementary_Data

## Data Availability

All data generated or analyzed in this study are available within the article. Additional information can be obtained from the corresponding authors upon reasonable request.
