# Supplementary material for: A photo-crosslinkable hyaluronic acid–collagen hydrogel maintains epithelial integrity and supports intestinal organoid stemness
Source: Regen Biomater. 2026 Aug 7;13:rbag171. doi: 10.1093/rb/rbag171 (PMC13641166; doi:10.1093/rb/rbag171)
Supplement: Supplementary file 1 [file rbag171_supplementary_data.zip › Supplementary_Figure_S1.docx]

Figure S1. Optimization of photo-crosslinking parameters and validation of progenitor cell maintenance across matrix formulations. (A) Frequency-dependent changes in storage (G') and loss (G") moduli for different RFP concentration and blue light irradiation (B) Comparison of final storage modulus values across different matrix formulations measured at 1 Hz. (C) Western blotting images of intestinal organoid cultured in various matrix formulations. (D) Relative protein level of Sox9 of intestinal organoid. Bars represent mean ± S.D. The * and # symbols indicate differences compared with the Matrigel and 0.005% 15s group at p < 0.05, respectively.
