# Supplementary material for: A photo-crosslinkable hyaluronic acid–collagen hydrogel maintains epithelial integrity and supports intestinal organoid stemness
Source: Regen Biomater. 2026 Aug 7;13:rbag171. doi: 10.1093/rb/rbag171 (PMC13641166; doi:10.1093/rb/rbag171)
Supplement: Supplementary file 1 [file rbag171_supplementary_data.zip › Supplementary_Figure_S2.docx]

Figure S2. Strain-dependent stress relaxation behavior of hydrogel matrices. Time-dependent stress relaxation curves under varying strain conditions (10%, 30%, and 60%) for (A) Matrigel, (B) Collagen, (C) Col2HA1, (D) Col3HA1, and (E) Col4HA1. Values represent mean ± SD (n = 3).
