# Supplementary material for: A photo-crosslinkable hyaluronic acid–collagen hydrogel maintains epithelial integrity and supports intestinal organoid stemness
Source: Regen Biomater. 2026 Aug 7;13:rbag171. doi: 10.1093/rb/rbag171 (PMC13641166; doi:10.1093/rb/rbag171)
Supplement: Supplementary file 1 [file rbag171_supplementary_data.zip › Supplementary Figure S3.docx]

Figure S3. Microtubule organization and epithelial polarity of intestinal organoids cultured in different matrices. Confocal immunofluorescence image of intestinal organoid stained for DAPI and α-tubulin. Continuous α-tubulin–positive epithelial structures were observed across conditions, demonstrating that the defined HA-collagen matrices support proper apical-basal polarity essential for epithelial function. Scale bar = 50 µm.
