# Supplementary material for: A photo-crosslinkable hyaluronic acid–collagen hydrogel maintains epithelial integrity and supports intestinal organoid stemness
Source: Regen Biomater. 2026 Aug 7;13:rbag171. doi: 10.1093/rb/rbag171 (PMC13641166; doi:10.1093/rb/rbag171)
Supplement: Supplementary file 1 [file rbag171_supplementary_data.zip › Supplementary Figure S4.docx]

Figure S4. Whole blot image of villin for the response about reviewer 3’s 3^rd^ question.
